# Comparison of the Mandibular Buccal Shelf Bone Thickness in Adolescents and Adults Using Cone Beam Computed Tomography

**DOI:** 10.7759/cureus.104393

**Published:** 2026-02-27

**Authors:** Meghna Mittal, Shubham Gupta, Vani Jairaj, Saraswati Chandra, Ishu Agarwal, Manoj K Sharma, Seema Gupta

**Affiliations:** 1 Department of Orthodontics, Kothiwal Dental College and Research Centre, Moradabad, IND; 2 Department of Orthodontics, Sri Venkateshwara Dental College and Hospital, Bangalore, IND; 3 Department of Orthodontics, Shree Bankey Bihari Dental College and Research Centre, Ghaziabad, IND

**Keywords:** adolescents, adults, buccal bone, cbct, cone beam computed tomography, mandible, mandibular buccal shelf thickness, molar

## Abstract

Introduction

The mandibular buccal shelf is a reliable site for extra-alveolar orthodontic miniscrew placement. The primary stability of temporary anchorage devices depends largely on cortical bone thickness and overall bone morphology. However, age-related variations in buccal shelf bone dimensions have not been fully established, particularly between adolescents and adults with similar growth patterns. The aim of the present study was to evaluate and compare the vertical and horizontal bone thickness of the mandibular buccal shelf in adolescent and adult populations using cone-beam computed tomography (CBCT).

Materials and methods

This retrospective cross-sectional study included 60 CBCT scans divided into two groups: adolescents (12-18 years) and adults (19-35 years), with 30 subjects in each group. Only individuals with average growth patterns and complete permanent dentition were included in the study. Vertical bone thickness was measured at 3, 5, and 7 mm apical to the cemento-enamel junction (CEJ) at three locations: mesial to the first molar, between the first and second molars, and distal to the second molar. Horizontal thickness was assessed at 6 mm and 11 mm apical to the CEJ at the corresponding sites. The measurements were performed bilaterally and averaged. Data were analyzed using mixed-model analysis of variance (ANOVA), with significance set at p<0.05.

Results

Vertical bone thickness increased with depth and showed significant site-related variations. Adults demonstrated significantly greater vertical thickness at deeper levels, particularly in the inter-radicular regions. Horizontal thickness also increased apically, with adults showing greater values at the mesial and inter-radicular sites. The region distal to the second molar consistently exhibited favorable dimensions in both the groups.

Conclusion

The thickness of the mandibular buccal shelf bone varies according to age, depth, and location. Adults demonstrate more favorable bone dimensions for miniscrew stability, particularly in the deeper and inter-radicular regions. CBCT-based individualized assessment is recommended for optimal extra-alveolar anchorage planning.

## Introduction

One of the biggest issues that clinicians worry about while moving teeth in orthodontics is anchorage loss. Although absolute anchorage has long been considered the ideal treatment, it is rarely achieved successfully. Absolute skeletal anchorage using miniscrews is widely used nowadays, as it not only improves orthodontic mechanics but also offers good patient acceptance and relatively low failure rates. Primary stability is vital for the successful placement of miniscrews [1]. Orthodontic miniscrews provide absolute anchorage and have become more commonly used in recent years for skeletal anchorage. The use of screw-type mini-implants, designed specifically for orthodontic anchorage, allows positioning in various locations owing to their small diameter and length [2]. They are reasonably priced, easy to insert and remove, can be loaded immediately or early, and provide sufficient anchorage support for non-compliant patients [3]. They are recommended for correcting deep bites, en masse retraction of anterior teeth, extrusion of impacted canines, and closure of extraction spaces [4,5]. The preferred anatomic sites for miniscrew implantation include the palate, lingual aspect of the maxillary alveolar process, retromolar region, and maxillary and mandibular buccal alveolar processes [4]. Cone-beam computed tomography (CBCT) has been widely used in studies to measure cortical bone thickness, identify optimal anatomic sites, and assess tissues at risk in various locations.

The location of the mini-implant is crucial for attaining absolute anchorage, with higher success rates in areas with greater cortical bone thickness. Mandibles generally have greater bone thickness than the maxilla. Recently, the mandibular buccal shelf has emerged as the preferred insertion site for orthodontic miniscrews [6]. Implants are situated buccal to the roots of the mandibular molars in the posterior mandibular body on both sides and can be placed next to the first molar, between the first and second molars, or near the second molar. Due to variations in buccal shelf bone thickness, this region offers superior quality compared to other sites, influenced by factors such as ethnicity, growth pattern, and malocclusion type [7,8]. Inserting miniscrews laterally to the first and second molars, approximately 5-7 mm below the alveolar crest, perpendicular to the occlusal plane, and parallel to the dental axis inclination minimizes interference from tooth displacement and reduces the risk of entering the mandibular canal.

CBCT has proven accurate for over a decade in measurements that are unattainable by previous imaging techniques. Unlike conventional CT, which uses a fan-shaped beam and higher radiation, CBCT employs a cone-shaped beam with a single pass around the patient in less than 70 seconds, reducing exposure to as little as one-sixth. It provides quick, low-radiation, 3D imaging with multiple slices [9]. This study aimed to evaluate and compare the bone thickness of the mandibular buccal shelf area in adolescent and adult populations with average growth patterns using CBCT.

## Materials and methods

Study design

This retrospective cross-sectional study was conducted in May 2023 at the Department of Orthodontics, Kothiwal Dental College and Research Centre, Moradabad, Uttar Pradesh, India, using CBCT records obtained over the preceding three years (June 2020 to March 2023). Ethical approval was obtained from the Institutional Ethics Review Board (KDCRC/IERB/04/2023/25 dated 07-04-2023) prior to the commencement of the study. All CBCT scans were retrieved from the archives of the Radiology Department, ensuring patient anonymity and compliance with ethical guidelines.

Sample selection

A total of 60 CBCT scans were selected and divided equally into two age groups: Group A (adolescents, aged 12-18 years) and Group B (adults, aged 19-35 years), with 30 scans per group. Participants were limited to those exhibiting average growth patterns, as determined by cephalometric analysis using parameters such as the mandibular plane angle (SN-MP: 32^0^ ± 5^0^), facial axis angle (90^0^ ± 3^0^), and lower facial height ratio (45% ± 3%). Only scans from individuals residing in Moradabad city were included to control for the regional variations. The sample size was calculated using G*Power software (version 3.1, Heinrich-Heine-Universität Düsseldorf, Düsseldorf, Germany). The calculation was based on the primary outcome of comparing mean buccal shelf cortical bone depth between two independent groups. Based on data from a previous study [10], an effect size of 0.5 was deemed clinically significant. To achieve 80% statistical power with an alpha error of 0.05, a minimum of 60 participants were required.

Inclusion and exclusion criteria

The inclusion criteria comprised CBCT scans of healthy individuals with average growth patterns, complete permanent dentition (excluding third molars), no history of orthodontic treatment, and high-quality scans with a field of view covering the bilateral mandibular posterior region. The exclusion criteria included scans showing craniofacial anomalies, systemic diseases affecting bone metabolism (e.g., osteoporosis and endocrine disorders), history of trauma or surgery in the mandibular region, presence of periodontal disease, or artifacts that obscured the buccal shelf area. Additionally, individuals with extreme growth patterns (high or low angle) or malocclusions requiring surgical intervention were excluded to maintain the focus on average growers.

Data collection and imaging protocol

CBCT scans were acquired using a standardized protocol with a Carestream CS 9300 machine (Carestream Dental LLC, Atlanta, GA, USA) set at 90 kVp, 4 mA, and a voxel size of 0.3 mm. The field of view was 10 x 10 cm, which encompassed the entire mandible. Scans were exported in DICOM format and analyzed using CS 3D Imaging Software (version 3.8.7; Carestream Dental LLC, Atlanta, GA, USA). For each scan, the mandibular buccal shelf was identified as the area buccal to the roots of the mandibular first and second molars. Measurements were performed bilaterally to account for symmetry, and the average value was used for the analysis.

Measurement procedure

The bone thickness of the mandibular buccal shelf was assessed in both vertical and horizontal dimensions using CBCT images. The region of interest was defined as the area buccal to the roots of the mandibular first and second molars. All reconstructed images were oriented in the axial, sagittal, and coronal planes to ensure standardized head positioning prior to measurement.

**Figure 1:**
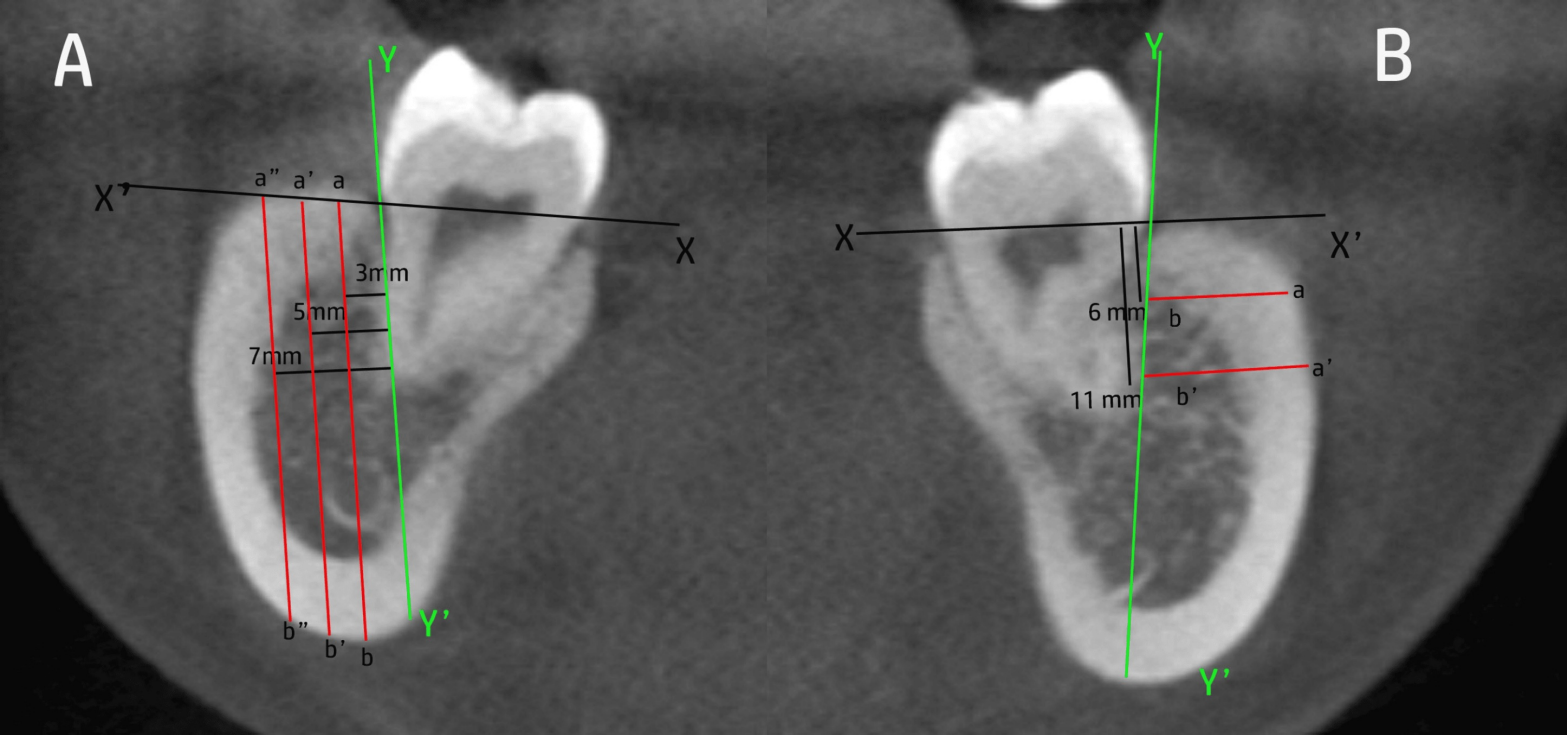
Cone-beam computed tomographic (CBCT)-based assessment of mandibular buccal shelf bone thickness The images shown are original CBCT patient images obtained from the study sample. Y–Y′ represents the long axis of the tooth along the root surface, X–X′ represents the reference line at the CEJ level. (A) Vertical thickness of the buccal shelf bone measured at 3 mm, 5 mm, and 7 mm from the buccal surface of the root. Point a denotes a point on the CEJ reference line and point b denotes a point on the inferior border of the mandible; distances a–b, a'–b′, and a''–b″ indicate vertical measurements taken at 3 mm, 5 mm, and 7 mm, respectively; (B) Horizontal thickness of the buccal shelf bone measured at 6 mm and 11 mm apical to the cementoenamel junction (CEJ). Point b represents a point on the root surface and point a represents a point on the buccal cortical plate; distances a–b and a'–b′ indicate horizontal alveolar bone thickness measured at 6 mm and 11 mm apical to the CEJ.

For vertical buccal shelf thickness, measurements were performed in the sagittal sections at three anatomical locations: mesial to the first molar, between the first and second molars (inter-radicular region), and distal to the second molar. At each site, measurements were taken at three different depths (3, 5, and 7 mm) apical to the cemento-enamel junction (CEJ) (Figure 1A).

Vertical thickness was defined as the linear distance from the buccal cortical plate to the inferior border of the mandibular body or basal bone along a line perpendicular to the occlusal plane [10].

For horizontal buccal shelf thickness, measurements were performed in the axial (transverse) sections at two levels: 6 and 11 mm apical to the CEJ (Figure 1B). At each level, measurements were obtained at the same three anatomical locations (mesial to the first molar, between the first and second molars, and distal to the second molar). Horizontal thickness was defined as the buccolingual distance from the outer buccal cortical plate to the lingual cortical boundary measured perpendicular to the long axis of the tooth [10].

All measurements were performed bilaterally, and the mean of the right and left sides was calculated for statistical analysis. To minimize measurement bias, all measurements were conducted by a single, calibrated investigator. Intra-observer reliability was assessed by repeating the measurements on 10 randomly selected CBCT scans after a two-week interval, and reliability was confirmed using the intraclass correlation coefficient (ICC >0.90).

Statistical analysis

Data were analyzed using the IBM SPSS Statistics for Windows, Version 25 (Released 2017; IBM Corp., Armonk, New York, United States). Descriptive statistics, including the mean and standard deviation, were calculated for the bone thickness in each group. Normality was tested using the Shapiro-Wilk test. A mixed model analysis of variance (ANOVA) was employed to compare alveolar bone thickness between adolescent and adult groups at different depths of bone and locations with mandibular molar teeth. Statistical significance was set at p<0.05.

## Results

The demographic characteristics of the study participants showed that adolescents had a mean age of 15.35 ± 4.36 years, while adults had a mean age of 25.85 ± 5.25 years. Both groups demonstrated equal gender distribution, with 15 female participants (50%) and 15 male participants (50%) in each group (Table 1).

**Table 1:** Demographic characteristics of the study groups Data presented in mean and standard deviation (SD) and frequency (%).

Parameter	Unit	Adolescent group	Adult group
Age (Years)	Mean ± SD	15.35 ± 4.36	25.85 ± 5.25
Females	n (%)	15 (50%)	15 (50%)
Males	n (%)	15 (50%)	15 (50%)

Vertical thickness of the buccal shelf area

At 3 mm from the root surface, adolescents demonstrated comparable or slightly higher mean bone thickness than adults across most sites. At the mesial aspect of the first molar, adolescents measured 16.42 ± 2.91 mm compared to 15.58 ± 2.61 mm in adults. Between first and second molars, values were 18.2 ± 1.8 mm in adolescents versus 19.06 ± 1.95 mm in adults. At the 5 mm level, notable differences emerged in the inter-radicular region between first and second molars, where adults showed substantially greater thickness (17.11 ± 1.9 mm) compared to adolescents (11.75 ± 4.29 mm). Similarly at 7 mm in the same region, adults maintained higher values (16.98 ± 1.43 mm) versus adolescents (11.13 ± 1.92 mm). These findings indicate that while vertical bone thickness at 3 mm is similar between groups, adolescents have thinner bone at 7 mm inter-radicular zones, suggesting continued bone deposition into adulthood that enhances structural support for the dentition (Table 2).

**Table 2:** Vertical bone thickness of the buccal shelf area (mm) at 3, 5, and 7 mm from the buccal surface of mandibular molar roots in adolescents and adults Values are expressed as mean ± standard deviation (SD), CI: confidence interval.

Vertical thickness of bone		Adolescent group		Adult group	
Distance	Location	Mean ± SD	95% CI of mean	Mean ± SD	95% CI of mean
At 3 mm	Mesial to the first molar	16.42 ± 2.91	14.34 - 18.50	15.58 ± 2.61	13.71 - 17.45
	Between the first and the second molars	18.20 ± 1.80	16.91 - 19.49	19.06 ± 1.95	17.66 - 20.46
	Distal to the second molar	19.63 ± 2.26	18.01 - 21.25	20.41 ± 2.00	18.98 - 21.84
At 5 mm	Mesial to the first molar	13.23 ± 3.42	10.79 - 15.67	13.51 ± 2.40	11.79 - 15.23
	Between the first and the second molars	11.75 ± 4.29	8.68 - 14.82	17.11 ± 1.90	15.75 - 18.47
	Distal to the second molar	19.54 ± 2.80	17.53 - 21.55	18.56 ± 1.17	17.72 - 19.40
At 7 mm	Mesial to the first molar	8.83 ± 2.84	6.80 - 10.86	12.19 ± 2.11	10.68 - 13.70
	Between the first and the second molars	11.13 ± 1.92	9.76 - 12.50	16.98 ± 1.43	15.96 - 18.00
	Distal to the second molar	18.15 ± 2.90	16.07 - 20.23	18.83 ± 1.17	17.99 - 19.67

Mixed-model ANOVA revealed significant main effects of location on vertical bone thickness at all three depths (p=0.001). At the 3 mm depth, no significant age group difference (p=0.695) or interaction (p=0.371) was observed. At 5 mm, significant effects were found for age group (p=0.043) and location × age group interaction (p=0.003). At 7 mm, both location (p=0.001) and age group (p=0.001) showed significant main effects, with significant interaction (p=0.003). Age-related changes were depth-dependent: while buccal shelf bone (3 mm) showed similar architecture between groups, and at 5-7 mm exhibited significant maturational differences, highlighting that mandibular bone maturation continues into adulthood with pronounced changes in deeper cancellous regions (Table 3). 

**Table 3:** Mixed-model analysis of variance (ANOVA) for vertical bone thickness at 3, 5, and 7 mm (location-based analysis) df = degrees of freedom, *Statistically significant at p<0.05.

Depth	Source of Variance	Sum of squares	df	F value	p-value
At 3 mm	Locations	166.73	2	18.47	0.001*
	Age group	1.07	1	0.16	0.695
	Location x Age group	9.20	2	1.02	0.371
At 5 mm	Locations	364.87	2	21.89	0.001*
	Age group	36.19	1	4.72	0.043*
	Location x Age group	112.65	2	6.76	0.003*
At 7mm	Locations	639.44	2	67.94	0.001*
	Age group	163.02	1	35.23	0.001*
	Location x Age group	66.85	2	7.10	0.003*

Significant main effects of distance were observed at all three locations (p=0.001). At the mesial aspect, a significant distance × age group interaction (p=0.001) was found without a main effect of age (p=0.416). Between the first and the second molars, significant main effects emerged for both distance (p=0.001) and age group (p=0.001), along with a significant interaction (p=0.001). Distal to the second molar, distance (p=0.001) and the interaction term (p=0.029) were significant, but age group alone was not (p=0.858). The inter-radicular region showed the most pronounced maturational changes with consistent age effects across all depths, reflecting ongoing skeletal maturation and functional adaptation (Table 4). 

**Table 4:** Mixed-model ANOVA for vertical bone thickness according to distance from buccal surface df = degrees of freedom, *Statistically significant at p<0.05.

Location	Source of variance	Sum of squares	df	F value	p-value
Mesial to the first molar	Distances (3,5 and 7 mm)	301.58	2	80.51	0.001*
	Age group	13.07	1	0.69	0.416
	Distances x Age group	47.30	2	12.63	0.001*
Between the first and the second molars	Distances (3,5 and 7 mm)	258.08	2	27.90	0.001*
	Age group	242.81	1	29.81	0.001*
	Distances x Age group	75.65	2	8.18	0.001*
Distal to the second molar	Distances (3,5 and 7 mm)	23.97	2	9.63	0.001*
	Age group	0.38	1	0.03	0.858
	Distances x Age group	9.77	2	3.93	0.029*

Horizontal thickness of the buccal shelf area

At 6 mm apical to CEJ, adults demonstrated significantly greater horizontal bone thickness on the mesial aspect of the first molar (3.28 ± 1.02 mm) compared to adolescents (1.92 ± 0.53 mm). Between the first and the second molars, adults also showed higher values (4.13 ± 1.13 mm) than adolescents (3.03 ± 0.63 mm). Conversely, adolescents exhibited greater thickness distal to the second molar (5.76 ± 0.87 mm) compared to adults (3.65 ± 0.95 mm). At 11 mm depth, adults maintained higher values mesially (5.06 ± 0.81 mm vs. 4.01 ± 1.08 mm) and inter-radicularly (5.97 ± 0.67 mm vs. 4.79 ± 0.71 mm). Distal thickness was comparable between groups (6.85 ± 0.58 mm in adults vs. 6.92 ± 1.16 mm in adolescents). Age-related horizontal bone expansion occured preferentially in mesial and inter-radicular regions, reflecting functional adaptation to masticatory forces (Table 5). 

**Table 5:** Horizontal alveolar bone thickness of the buccal shelf area (mm) at 6 mm and 11 mm apical to the cemento-enamel junction (CEJ) of mandibular molars Values are expressed as mean ± standard deviation (SD), CI: confidence interval.

Horizontal thickness of bone		Adolescent group		Adult group	
Distance	Location	Mean ± SD	95% CI of mean	Mean ± SD	95% CI of mean
At 6 mm	Mesial to the first molar	1.92 ± 0.53	1.54 - 2.30	3.28 ± 1.02	2.55 - 4.01
	Between the first and the second molars	3.03 ± 0.63	2.58 - 3.48	4.13 ± 1.13	3.32 - 4.94
	Distal to the second molar	5.76 ± 0.87	5.14 - 6.38	3.65 ± 0.95	6.44 - 7.26
At 11 mm	Mesial to the first molar	4.01 ± 1.08	3.24 - 4.78	5.06 ± 0.81	2.97 - 4.33
	Between the first and the second molars	4.79 ± 0.71	4.28 - 5.30	5.97 ± 0.67	4.48 - 5.64
	Distal to the second molar	6.92 ± 1.16	6.09 - 7.75	6.85 ± 0.58	7.70 - 9.20

At 6 mm apical to CEJ, mixed model ANOVA revealed significant main effects of location (p=0.001) and age group (p=0.001), with no significant interaction (p=0.793). At 11 mm depth, the location showed a significant main effect (p=0.001), while age group alone was not significant (p=0.142). However, a significant location × age group interaction emerged at 11 mm (p=0.003). Age-related differences were depth-dependent: at the shallower 6 mm level, adults consistently exhibited greater bone thickness across all locations, while at the deeper 11 mm level, maturational patterns varied by location, suggesting site-specific trabecular remodeling (Table 6).

**Table 6:** Mixed-model ANOVA for horizontal bone thickness at 6 mm and 11 mm df = degrees of freedom, *Statistically significant at p<0.05.

Depth	Source of variance	Sum of squares	df	F value	p-value
At 6 mm	Locations	147.42	2	147.00	0.001*
	Age group	21.00	1	20.03	0.001*
	Location x Age group	0.23	2	0.23	0.793
At 11 mm	Locations	157.85	2	115.34	0.001*
	Age group	3.46	1	2.36	0.142
	Location x Age group	9.26	2	6.77	0.003*

Mixed-model ANOVA demonstrated significant main effects of distance at all the three locations (p=0.001). At the mesial aspect, a significant distance × age group interaction (p=0.001) was observed without a main effect of age (p=0.192). Between the first and the second molars, the interaction was significant (p=0.034), while age group approached significance (p=0.052). Distal to the second molar, significant main effects emerged for both distance (p=0.001) and age group (p=0.003), with no significant interaction (p=0.215). The distal region showed consistent age effects across both depths indicating uniform maturational expansion, while mesial and inter-radicular regions exhibited complex patterns where age differences depended on the specific depth measured (Table 7). 

**Table 7:** Mixed-model ANOVA for horizontal bone thickness according to distance from CEJ CEJ: cemento-enamel junction; df: degrees of freedom; *Statistically significant at p<0.05.

Location	Source of variance	Sum of squares	df	F value	p-value
Mesial to the first molar	Distances (6 and 11 mm)	15.13	1	45.20	0.001*
	Age group	2.50	1	1.84	0.192
	Distances x Age group	7.40	1	22.10	0.001*
Between the first and the second molars	Distances (6 and 11 mm)	18.09	1	55.17	0.001*
	Age group	4.69	1	4.32	0.052
	Distances x Age group	1.72	1	5.25	0.034*
Distal to the second molar	Distances (6 and 11 mm)	19.04	1	64.90	0.001*
	Age group	17.16	1	11.60	0.003*
	Distances x Age group	0.48	1	1.65	0.215

Overall, both vertical and horizontal measurements indicated that mandibular alveolar bone maturation continues into adulthood, with the most pronounced differences observed in the deeper and inter-radicular regions. These findings highlight the depth- and site-specific nature of skeletal development in the posterior mandible.

## Discussion

The present study demonstrated a clear depth- and site-dependent maturation of the mandibular buccal shelf bone. While superficial vertical thicknesses were similar between adolescents and adults, the latter showed greater thickness at deeper vertical levels and at inter-radicular (between the first and the second molar) locations. Horizontally, adults tended to have thicker bone mesially and inter-radicularly, while the distal regions showed more variable age patterns. These results fit a biologically plausible picture that cortical and trabecular bones continue to remodel and consolidate with age and functional loading; therefore, the basal cancellous regions and inter-radicular supports mature later than the thin superficial cortex.

Several previously published CBCT studies have reported findings that align with ours. Farahania et al. [11] found that the distal region of the second molar consistently provided adequate width for miniscrew fixation and highlighted age differences in some buccal shelf sites, which is consistent with our observation of pronounced adult advantage at deeper and inter-radicular levels. Similar results were obtained by Abhijith et al. [12] and Escobar-Correa et al. [13]. A buccal bone thickness of no less than 5 mm (accounting for 1.7 mm for root safety clearance, 1.6 mm for the diameter of the screw, and 1.7 mm for the safety clearance of the cortical buccal bone) was identified as a secure benchmark for the placement of miniscrews [10].

Aleluia et al. [14] evaluated the mandibular buccal shelf using CT imaging to determine its suitability for orthodontic miniscrew anchorage, analyzing the influence of side, gender, and skeletal pattern. They found that bone thickness increased in the posterior and apical directions, with the region distal to the second molar showing the most favorable dimensions for miniscrew placement. Skeletal pattern significantly affected bone morphology, with Class III and hypodivergent patients exhibiting greater bone thickness, whereas side and sex differences were minimal. Derton et al. [15] conducted a CBCT-based study to evaluate the anatomical bone characteristics of the mandibular buccal shelf (buccal step) as a site for orthodontic mini-screw placement. They reported that bone thickness increased toward the posterior and apical regions, with the area distal to the second molar offering the most favorable dimensions for a stable miniscrew insertion. The authors emphasized the importance of an individualized CBCT assessment to optimize primary stability and minimize surgical risks. Collectively, these studies and our results underline that the posterior mandible near the second molar is often the most robust site for extra-alveolar anchorage in adults, whereas adolescents may require more cautious planning.

Mechanistically, the observed patterns can be explained by functional adaptations. Masticatory load and occlusal forces are larger and more stable in adults than in children. Over time, these mechanical stimuli promote cortical thickening and trabecular reorganization, particularly in the load-bearing posterobasal regions. The inter-radicular region between the first and the second molars is subject to complex force vectors and tends to accrue supportive bone with age, which explains the larger adult means and significant age × location interactions observed [16,17].

Clinically, these findings have practical implications for the selection and placement of temporary anchorage devices (TAD). First, in adults, the deeper inter-radicular and mesial zones (particularly near the second molar) appear to offer greater bone stock and therefore improved primary stability, supporting the clinician’s choice of longer or slightly wider miniscrews when anatomy allows. Second, in adolescents, the relative thinning at the deeper inter-radicular levels suggests caution: avoid overconfident insertion depths and check root proximity carefully with CBCT to reduce the risk of root contact or perforation. Third, the variable distal second molar behavior, sometimes showing greater thickness in adolescents at specific horizontal levels, reminds us that “one size fits all” rules are unwise; individualized CBCT mapping remains the safest approach when planning extra-alveolar miniscrews. In practical terms, the plan should be inserted at levels and angulations that maximize cortical engagement (for primary stability) while preserving at least a 1-2 mm safety margin from the tooth roots and the mandibular canal [18].

Beyond TADs, the data are relevant to other clinical decisions: implant placement in the posterior mandible, planning corticotomies, or anticipating the risk of fenestration during large buccal movements. For orthodontists who treat mixed-age cohorts, this study provides evidence to prefer slightly more conservative mechanics in younger patients or to select alternative anchorage sites when CBCT shows insufficient bone at the desired site.

However, the limitations of this study temper our enthusiasm. This study was retrospective and cross-sectional; therefore, it could not prove a developmental trajectory within individuals. The sample was drawn from a single geographic region and included only average-growth patients, which limits the generalizability to other ethnicities or extreme vertical growers. Finally, functional variables (chewing forces and parafunction) and systemic bone health were not considered, which may have influenced local bone morphology.

## Conclusions

This study concluded that mandibular buccal shelf bone thickness demonstrates significant age-, depth-, and site-specific variations. Adults exhibited greater vertical thickness at deeper levels and increased horizontal thickness in the mesial and interradicular regions than adolescents, indicating continued skeletal maturation into adulthood. Bone thickness increased apically and posteriorly, with the region distal to the second molar consistently providing the most favorable dimensions for extra-alveolar miniscrew placement. These findings support careful CBCT-based evaluation before TAD insertion, particularly in adolescents, to optimize primary stability and reduce the risk of complications. The mandibular buccal shelf remains a reliable anchorage site when anatomical variations are considered.
